# Hand washing behavior and associated factors in Vietnam based on the Multiple Indicator Cluster Survey, 2010–2011

**DOI:** 10.3402/gha.v9.29207

**Published:** 2016-02-29

**Authors:** Kien Gia To, Jong-Koo Lee, You-Seon Nam, Oanh Thi Hoang Trinh, Dung Van Do

**Affiliations:** 1 Faculty of Public Health, University of Medicine and Pharmacy, Ho Chi Minh City, Vietnam; 2 JW LEE Center for Global Medicine, College of Medicine, Seoul National University, Seoul, Republic of Korea; 3 Department of Family Medicine, Seoul National University College of Medicine, Seoul, Republic of Korea

**Keywords:** hand washing, hand hygiene, public health practice, communicable disease control, Vietnam, community survey

## Abstract

**Background:**

Handwashing is a cost-effective way of preventing communicable diseases such as respiratory and food-borne illnesses. However, handwashing rates are low in developing countries. Target 7C of the seventh Millennium Development Goals was to increase by half the proportion of people with sustainable access to safe drinking water and basic sanitation by 2015. Studies have found that better access to improved water sources and sanitation is associated with higher rates of handwashing.

**Objective:**

Our goal was to describe handwashing behaviour and identify the associated factors in Vietnamese households.

**Design:**

Data from 12,000 households participating in the Vietnam Multiple Indicator Cluster Survey 2011 were used. The survey used a multistage sampling method to randomly select 100 clusters and 20 households per cluster. Self-administered questionnaires were used to collect data from a household representative. Demographic variables, the presence of a specific place for handwashing, soap and water, access to improved sanitation, and access to improved water sources were tested for association with handwashing behaviour in logistic regression.

**Results:**

Almost 98% of households had a specific place for handwashing, and 85% had cleansing materials and water at such a place. The prevalence of handwashing in the sample was almost 85%. Educational level, ethnicity of the household head, and household wealth were factors associated with handwashing practice (*p*<0.05). Those having access to an improved sanitation facility were more likely to practise handwashing [odds ratio (OR)=1.69, 95% confidence interval (CI): 1.37–2.09, *p*<0.001], as were those with access to improved water sources (OR=1.74, 95% CI: 1.37–2.21, *p*<0.001).

**Conclusions:**

Households with low education, low wealth, belonging to ethnic minorities, and with low access to improved sanitation facilities and water sources should be targeted for interventions implementing handwashing practice. In addition, the availability of soap and water at handwashing sites should be increased and practical teaching programs should be deployed in order to increase handwashing rates.

## Background

Hands are not only an indispensable tool used for daily activities but also a vector for spreading infection. Washing hands is an effective strategy for preventing the spread of many diseases. Evidence shows that washing hands halves the risk of pneumonia and diarrhoea, which are two of the leading causes of deaths worldwide in children under 5 years old (1). A study that followed a cohort of one-to-eleven-month-old children in Afghanistan showed that maternal handwashing with soap reduced the risk of diarrhoea in children by 15% (2). A systematic review of 17 studies – of which seven included interventions, six had case-control designs, two were cohort studies, and another two were cross-sectional studies – confirmed that handwashing reduces the risk of diarrhoea by 50% and saves millions of lives (3). A more recent Cochrane review also showed that handwashing promotion reduced diarrhoea episodes by 30% in both high-income countries and in communities found in low- and middle-income countries (4). A randomised controlled trial showed that bacteria were found in 44% of hands; this figure was reduced to 23% after washing hands with water alone and to 8% after washing hands with water and soap (5). Washing hands with soap before food preparation has been shown to reduce the risk of food-borne diseases (6).

Although handwashing with soap and water is a simple and efficient method for reducing the risk of infectious diseases (5–7), a large number of people do not wash their hands regularly or do not know how to wash their hands properly (6). Various factors are found to be associated with handwashing. One study showed that the frequency of handwashing was higher in females compared with males (8) and was positively associated with higher educational levels (8, 9). The availability of water sources in the house and the condition of household sanitation facilities have been found to be associated with handwashing behaviour (9).

Handwashing behaviour can be assessed using questionnaires, by handwashing demonstration, by direct observation, or by indirect observation. However, there is no consensus on a gold standard for identifying handwashing behaviour (10). Some argue that questionnaires produce higher handwashing rates and that people tend to over-report because this is seen as a desirable behaviour (10, 11). A study that compared the rates of handwashing behaviour assessed by questionnaires, direct observation, indirect observation, handwashing demonstration, and pocket voting showed that handwashing behaviour assessed by handwashing demonstration had the lowest rate (11). Direct observation of handwashing behaviour at recommended times, such as after using the toilet, before eating, or before feeding children could be a more reliable way of representing true handwashing rates, but such an approach would be challenging to establish on a national scale (7). Moreover, handwashing behaviour is affected by the characteristics, skill, and presence of the observer (10, 11). However, indirect observation, based on the presence of a specific place for hand washing in households and the presence of cleansing materials and water at the time of the observation, is one way of measuring handwashing rates (7, 11, 12).

One study found that soap and water are more likely to be available in households that have a specific place for handwashing (12). The availability of water and soap is strongly associated with higher prevalence of handwashing behaviour (11, 12). Handwashing is more likely to be practised in households that have a specific place for handwashing with availability of water and soap (11, 12). Studies have shown that the availability of water at the usual place for handwashing after defecation (13) and availability of soap when handwashing (12) were factors associated with a reduction in diarrhoeal or respiratory diseases in children (14). Another study showed that observation and self-reported questionnaires gave similar outcomes for handwashing with soap in Vietnamese schoolchildren (15).

Nonetheless, there is little information regarding handwashing behaviour among the Vietnamese. One study showed a low rate of handwashing with soap in rural schoolchildren in northern Vietnam (15). In that study, handwashing stations were not available or did not work in 66% of the schools (15). The study also found that those children with low school grades and from ethnic minorities were less likely to wash their hands with soap (15). The Vietnam Scaling Up Handwashing Behaviour study conducted a baseline survey of 3,150 households to collect data on the characteristics of household members, accessibility to handwashing facilities, handwashing behaviour, the prevalence of diarrhoea and respiratory infection in children, and child growth and development. The findings showed that 80% of households had a specific place for handwashing with the presence of soap and water (16). That study applied multistage random sampling to select 140 communes from two northern districts and one southern district, but districts located in the middle of Vietnam were not represented, thus limiting the generalisability of the results.

Vietnam is a developing country, with 54 ethnic groups, of which the Kinh account for 86% of the total population of 86 million (17). Vietnam has 58 provinces, 4 municipalities, and a capital city. It is divided into six geographic regions, and each region can be subdivided into rural and urban living areas. Our study used data from a large representative national sample to assess the handwashing behaviour of Vietnamese households and associated factors, taking into account different regional and socio-economic conditions.

## Methods

### Data collection and study design

This study assessed handwashing behaviour and its associated factors using data from the Vietnam Multiple Indicator Cluster Survey (MICS), which is an ongoing survey that has been conducted by the General Statistics Office of Vietnam and the United Nations Children's Fund since 2000. The MICS collects information on children, women, and key indicators related to the Millennium Development Goals using a household questionnaire, which is administered to a household representative. The survey has now been conducted four times in Vietnam. However, MICS4 (2010–2011) was the only dataset used here because the surveys conducted in the other 3 years did not include data on handwashing behaviour (18).

The sample size of 12,000 households was estimated and selected using a multistage sampling method, with clusters as the primary sampling unit (PSU). A systematic probability proportional to size sampling method was applied to select 100 clusters for each region, stratified by rural and urban areas. Random systematic sampling was applied to select 20 households in each cluster (7).

### Independent variables

Demographic information included the six Vietnamese regions, living areas, educational level, ethnicity, religion of the household head, and household wealth index. Other independent variables were the availability of an improved sanitation facility and improved water sources. The six Vietnamese regions were the Red River Delta; Northern Midland and Mountain Areas; North Central Area and Central Coastal Areas; Central Highlands; South East; and Mekong River Delta. Living areas were indicated as rural or urban. Educational levels of household heads were classified as none, primary school, junior high school, senior high school, and tertiary or higher level of education. Ethnicity was divided into Kinh/Hoa and other minority groups. Religion was grouped into Buddhism, Cao Dai, Hoa Hao, Catholic, Protestant, and other religion or no religion. A household wealth index was classified into five equal quintiles, with the first indicating the poorest and the fifth the richest. The wealth index (provided by the MICS) was derived using information on water source, toilet facility, housing, fuel types for cooking, electricity, bank account, durable goods (such as radio, TV, refrigerator, fixed telephone, watch, mobile phone, bicycle, motorcycle, boat with motor, car), and animals (such as buffalo, cattle, horse, donkey, goat, sheep, chicken, pig) (7).

An *improved sanitation facility* was defined as ‘one that hygienically separates human excreta from human contact’ (18). This includes ‘flush or pour flush toilets flowing to a piped sewer system, septic tank, or latrine; ventilated improved pit latrine, pit latrine with slab, and composting toilet’ (19). By observing the type of toilet facility used by each household, a household was classified as having or not having an improved sanitation facility.

A household was considered to be using *improved water sources* if it used ‘piped water (into dwelling, compound, yard or plot, piped to neighbour, public tap/standpipe), tube well/borehole, protected well, protected spring, and rain water collection’ for drinking purposes. If bottled water was used, the households must have had to use any of the improved water sources listed above for other purposes such as cooking and washing hands, to be considered as using improved water sources (18, 19).

#### Dependent variable

The dependent variable *handwashing behaviour* was derived as follows. A household was considered to be practising handwashing if 1) there was a specific place for washing hands; and 2) cleansing materials (i.e. soap) and water were available at that specific place. This was assessed by indirect observation when interviews with the household representative were conducted (18).

### Statistical analysis

Data were analysed using STATA12 based on the instructions from the United Nations Development Programme (20). Only households with completed questionnaires were included.

Survey sampling weights were applied to account for the complex survey design. The sampling fraction and the non-response rate adjustment were used to calculate the sample weights. The sample weights are the inverse of the sampling fractions. ‘The sampling fraction (f_hi_) for the i-th sample PSU in the h-th stratum is the probabilities of selection at every stage in each sampling stratum: f_hi_=p_1h_xp_2h_xp_3h_ where p_shi_ is the probability of selection of the sampling unit at stage s for the i-th sample PSU in the h-th sampling stratum’ (7). Because the estimated number of households in each cluster in the sampling frame was different from the updated number of the households in the cluster from the listing, individual sampling fractions were calculated for households in each sample cluster. The individual sampling fractions for households in each PSU is the first stage probability of selection of cluster in that particular sampling stratum and the second stage probability of selection of a household in the sample cluster (7). The non-response rate adjustment was ‘the number of occupied households listed in stratum h divided by the number of interviewed households in stratum h’ (7).

Households (percentages) having a specific place for handwashing, having water and cleansing materials, and practising handwashing were estimated by household characteristics.

Logistic regression identified the association between independent variables, including regions, living areas, household head characteristics (such as ethnicity, religion, and educational level), household wealth index, improved sanitation facilities, and improved water sources, with handwashing behaviour as the dependent variable. The choice of independent variables was informed by previous findings reported in the literature (9, 12, 15, 21, 22). The candidate variables were entered into a multivariable logistic regression model using a stepwise backward method with a threshold *p*-value set at 0.075 for removing a variable (23). Crude odds ratios (ORs) and adjusted ORs (aORs) were reported. All *p*-values were two-sided and considered significant if greater than 0.05. Taylor series linearization methods were used for variance estimation.

## Results

Among the 12,000 estimated households, 11,642 households were present at the time of the survey. The response rate was 99.8% (11,614 out of 11,642 completed the survey). There were 28 cases with missing information on education and 2 cases with missing information on religion. Those records were excluded from the analysis.

Table 1 shows the percentages of households with handwashing behaviour (so defined). Table 1 also gives the odds of handwashing behaviour by household characteristics. Almost all households (97.9%) had a specific place for handwashing, whereas water and cleansing materials at the specific place for handwashing were available in 85.2% of households. The percentage of handwashing behaviour was estimated at 84.7%. Almost all households (92.6%) were using an improved water source but only a 73.5% had an improved sanitation facility.

**Table 1:** The percentage of households having water and soap and a specific place for handwashing and the association between household characteristics with handwashing behaviour, MICS4, 2010–2011 (*N*=11,614)

		Place for handwashing	Water and cleansing materials	Handwashing behaviour			
				
Variables	Total	available (%)	available (%)	(%)	OR	*p*	95% CI
Region							
Red River Delta	22.4	98.8	92.0	91.7	1		
Northern Midlands and Mountain Area	15.8	99.3	81.2	80.8	0.38	<0.001	0.24–0.62
Northern Central Coastal Area	21.7	97.8	79.8	79.4	0.35	<0.001	0.23–0.53
Central Highlands	5.2	98.7	81.7	81.5	0.40	<0.001	0.26–0.63
South East	16.1	96.4	90.2	89.9	0.81	0.371	0.52–1.28
Mekong River Delta	18.8	97.0	83.2	82.4	0.43	<0.001	0.29–0.63
Living area							
Rural	70.3	98.3	82.6	82.2	1		
Urban	29.7	97.1	91.2	90.7	2.11	<0.001	1.67–2.65
Educational level of household head^a^							
None	6.0	95.7	65.4	64.8	1		
Primary school	25.2	97.6	79.1	78.5	2.00	<0.001	1.57–2.52
Junior high school	39.4	98.3	86.5	86.1	3.36	<0.001	2.60–4.35
Senior high school	16.4	98.0	91.0	90.7	5.31	<0.001	3.87–7.29
Tertiary and higher	13.0	98.5	94.8	94.5	9.34	<0.001	6.48–13.46
Household wealth index							
Poorest percentile	20.0	98.1	68.4	67.8	1		
2nd percentile	20.4	98.5	83.3	83.0	2.31	<0.001	1.87–2.85
3rd percentile	20.7	98.9	87.6	87.5	3.31	<0.001	2.58–4.25
4th percentile	20.0	96.9	91.9	91.0	4.80	<0.001	3.70–6.24
Richest percentile	18.8	97.1	95.2	95.1	9.14	<0.001	6.77–12.35
Ethnicity of household head							
Other minorities	10.1	98.2	66.4	65.8	1		
Kinh/Hoa	89.9	97.9	87.3	86.9	3.43	<0.001	2.50–4.73
Religion of household head^b^							
No religion	70.9	98.2	86.3	85.9	1		
Buddhist	19.2	97.4	81.6	81.1	0.71	0.002	0.56–0.88
Cao Dai	18.3	96.4	82.1	80.3	0.67	0.022	0.47–0.94
Hoa Hao	12.3	92.8	71.5	70.9	0.40	<0.001	0.23–0.69
Catholic	6.1	97.7	88.5	88.3	1.24	0.182	0.90–1.71
Protestant	5.4	97.1	71.7	70.4	0.39	0.013	0.19–0.82
Other religion	1.3	100.0	92.4	92.4	2.00	0.521	0.24–16.60
Improved sanitation facilities							
No	26.5	97.4	72.8	72.1	1		
Yes	73.5	98.1	89.6	89.3	3.23	<0.001	2.68–3.90
Improved water source							
No	7.4	97.3	64.3	63.6	1		
Yes	92.6	98.0	86.8	86.4	3.64	<0.001	2.81–4.71
Total	100	97.9	85.2	84.7			

MICS4, Multiple Indicator Cluster Survey, fourth wave; OR, odds ratio; CI, confidence interval; p-value significant at 0.05; all used logistic regression, except where otherwise stated.

a Twenty-eight cases missing information about educational level of household head were excluded.

b Two cases missing information about religion of household head were excluded.

In Table 1, logistic regression showed that, compared with the Red River Delta Region, the odds of washing hands in other regions were lower. The differences were statistically significant except when comparing the odds of washing hands in the South East Region with the Red River Delta Region (*p*=0.371).

The percentage of households living in urban areas was 29.7%. People living in urban areas were more than twice as likely to wash their hands compared to those who lived in rural areas [OR=2.11, 95% confidence interval (CI): 1.67–2.65].

The Kinh and Hoa ethnic groups represented 89.9% of the study sample and were more likely to wash their hands compared to other minor ethnic groups (OR=3.43, 95% CI: 2.50–4.73). The households that used an improved sanitation facility were more than three times as likely to wash their hands compared to those that did not use an improved sanitation facility (OR=3.23, 95% CI: 2.68–3.90). The households that used improved water sources were more than three and a half times as likely to wash their hands compared with those that did not (OR=3.64, 95% CI: 2.81–4.71).

The multivariable logistic regression model identified factors associated with handwashing behaviours. Stepwise backward methods with a threshold *p*-value of 0.075 removed region, living area, and religion from the full model, as their *p*-values were high (>0.425). The final model included educational level and ethnicity of the household head, household wealth index, improved sanitation facility, and improved water sources (Table 2).

**Table 2:** Multivariable logistic regression of the association between handwashing behaviour and associated factors, MICS4, 2010–2011 (*N*=11,580)

Associated factors	aOR	*p*	95% CI
Educational level of household head			
None	1		
Primary school	1.56	<0.001	1.24–1.95
Junior high school	1.96	<0.001	1.56–2.47
Senior high school	2.41	<0.001	1.77–3.27
Tertiary and higher	3.32	<0.001	2.32–4.75
Household wealth index			
Poorest percentile	1		
2nd percentile	1.60	<0.001	1.32–1.93
3rd percentile	1.88	<0.001	1.47–2.41
4th percentile	2.24	<0.001	1.68–2.97
Richest percentile	3.52	<0.001	2.53–4.88
Ethnicity of household head			
Other minorities	1		
Kinh and Hoa	1.45	0.015	1.07–1.97
Improved sanitation facility			
No	1		
Yes	1.69	<0.001	1.37–2.09
Improved water sources			
No	1		
Yes	1.74	<0.001	1.37–2.21

Multivariable logistic regression with backward method.

MICS4, Multiple Indicator Cluster Survey, fourth wave; aOR, adjusted odds ratio; CI, confidence interval.

There was a positive association between educational level of household heads and handwashing. The higher the educational level, the more likely it was that household members washed their hands, and the odds gradually increased depending on educational level. For instance, if the household head had a primary school educational level, households were 1.66 times more likely to wash their hands (aOR=1.66, 95% CI: 1.24–1.95, *p*<0.001) compared to households where household heads had no education. Households where household heads had a tertiary or higher educational level were more than three times as likely to wash their hands (aOR=3.32, 95% CI: 2.32–4.75, *p*<0.001) compared to households where household heads had no education.

The household wealth index showed similar results. When comparing the poorest quintile to the rest of the quintiles, households in the second quintile were 1.6 times more likely to wash their hands (aOR=1.6, 95% CI: 1.32–1.93, *p*<0.001), whereas people in the richest quintiles were more than three times (aOR=3.52, 95% CI: 2.53–4.88, *p*<0.001) as likely to wash their hands.

The Kinh and Hoa were almost one and a half times more likely to wash their hands compared to other minorities (aOR=1.45, 95% CI: 1.07–1.97, *p*=0.015). Households using improved sanitation facilities and improved water sources were also more likely to wash their hands (aOR=1.69, 95% CI: 1.37–2.09, *p*<0.001, and aOR=1.74, 95% CI: 1.37–2.21, *p*<0.001, respectively). Moreover, when interactions were tested between *improved sanitation facilities* and *improved water sources* with handwashing behaviour, no statistically significant interaction was found (*p*=0.802, data not shown).

## Discussion

This study used data from the MICS to determine the prevalence of handwashing behaviour and identify the factors related to handwashing behaviour in Vietnam. Our findings showed that 84.7% of the households practised handwashing behaviour, which is similar to the prevalence of 80.8% reported in a previous study (16). Other findings in this study similar to those of previous studies were that handwashing behaviour was positively correlated with higher education (9) and higher household wealth (16). Furthermore, our study was also consistent with the results of a previous study showing that households with limited access to water and sanitation facilities were less likely to wash their hands (9, 12).

A study in Kenya showed that areas with a high density of Muslim people had a higher proportion of handwashing (9). In our study, religion of the household head showed some association with handwashing behaviour in the bivariate analysis (e.g. compared with people with no religion, Hoa Hao people were significantly less likely to wash hands); however the association was not significant in the multivariable regression. People of Muslim religion accounted for less than 1% in the study population and were spread across diverse geographic areas (17), making it difficult to draw comparisons on this basis.

Regarding the ethnic groups in Vietnam, the Kinh represent 86% and the Hoa represent 1% of the total population (17). As in the MICS 2010–2011, the Kinh and Hoa were grouped together and compared with ethnic minorities, because these two groups had similar living standards (7). There was a higher rate of handwashing in the Kinh and Hoa people compared with other ethnic minorities. This can in part be explained by the fact that ethnic minorities had lower economic conditions (OR=0.08, 95% CI: 0.06–0.11, data not shown) and lower accessibility to improved sanitation facilities (OR=0.26, 95% CI: 0.19–0.35, data not shown) and improved water sources (OR=0.11, 95% CI: 0.07–0.16, data not shown) compared to the Kinh and Hoa. This accords with the results of one study conducted in northern rural Vietnam, which showed that not only ethnicity but also economic conditions affected the handwashing behaviour of schoolchildren belonging to ethnic minorities (15). Another study found that wealthier households were more likely to have soap and water in the house compared with poorer households and that the availability of soap and water increases the rate of handwashing (12). This finding suggests that interventions should increase the availability of soap and water facilities in the households of minor ethnicities with low wealth.

Nevertheless, an evaluation of primary schoolchildren from minor ethnic groups stressed the importance of teaching programmes rather than simply investing in handwashing facilities such as water and soap (24). Thus, besides providing water and soap for handwashing, school-based educational programs should be deployed, as handwashing behaviour is learned and consolidated during childhood (6). Teachers can effectively transfer information into children, who can then improve handwashing behaviours within their families (25).

A systematic review showed that hospital nurses can also educate and improve handwashing behaviours of patients, who will keep practising the learned behaviours even after being discharged (25). By the same token, school-based interventions or hospital-based interventions will be more effective if community-based interventions are conducted at the same time (24). As an example, mass media campaigns can increase the prevalence of handwashing in communities (26). However, evaluation of these community-based interventions is challenging and their effectiveness is wide ranging (25). Even so, health education programmes should be deployed at the school, hospital, and community levels in order to promote handwashing behaviour.

### Limitations of the study

This study has some limitations. Although the MICS has been conducted every 5 years since 2000 in Vietnam, data on handwashing were collected for the first time in the MICS 2010–2011 and therefore it was not possible to estimate the trends in handwashing behaviour over time. Another limitation relates to the measurement of handwashing behaviour in the MICS. Indirect observation has its limitations, but it is still a reliable method for handwashing assessment (7, 11, 12).

### Strengths of the study

The strengths of our study are that the MICS is a large national survey that is representative of all regions in Vietnam. The MICS has been conducted for some years and the procedures have been tested and standardised to ensure high quality data are collected.

## Conclusions

The study showed that the overall percentage of handwashing was almost 85%. In order to promote handwashing behaviour, availability of soap and water at the sites for handwashing should be increased and, more importantly, practical teaching programmes should be deployed. Educational level and ethnicity of the household head, household wealth index, and access to improved sanitation facilities and improved water sources were all associated with handwashing behaviour. These factors should be taken into account when designing interventions to help ensure that resources are directed to those most in need, such as people living in disadvantaged areas and of low socio-economic status.
